# Pharmacogenomics and genetic ancestry in Colombia: a study on all variant drug annotations of PharmGKB

**DOI:** 10.3389/fphar.2025.1636451

**Published:** 2025-08-29

**Authors:** Andy A. Acosta-Monterrosa, Kevin Fernando Montoya-Quintero, Johana Galván-Barrios, Indiana Luz Rojas Torres

**Affiliations:** ^1^ Faculty of Exact and Natural Sciences, Universidad de Cartagena, Cartagena, Colombia; ^2^ Center for Meta-Research and Scientometrics in Biomedical Sciences, Barranquilla, Colombia; ^3^ Facultad de Ciencias para la Salud, Universidad de Manizales, Manizales, Colombia; ^4^ Department of Health Sciences, Biomedical Scientometrics and Evidence-Based Research Unit, Universidad de la Costa, Barranquilla, Colombia; ^5^ Facultad de Ciencias de la Salud, Centro de Investigaciones en Ciencias de la Vida, Universidad Simón Bolívar, Barranquilla, Colombia

**Keywords:** pharmacogenomic variants, precision medicine, pharmacogenetics, genomics, Colombia

## Abstract

**Background:**

To generate an ancestry-resolved pharmacogenomic (PGx) landscape for Colombia by integrating all PharmGKB variant-drug annotations with local allele-frequency data, thereby quantifying inter-ancestry differences of clinical relevance and exposing evidence gaps that hinder equitable precision medicine.

**Methods:**

We conducted a cross-sectional analysis of 4,462 PharmGKB variant annotations (1994–2024), retaining 1,216 significant single-nucleotide polymorphisms (SNPs) reported in 552 studies. Allele frequencies were extracted for five Colombian populations: two predominantly African (Palenque [PLQ], Chocó [CHG]) and three predominantly European (ATQCES, ATQPGC, CLM), from the CÓDIGO database. Spearman correlations compared population-specific PGx profiles; SNPs with >25 percentage-point frequency differentials were tabulated.

**Results:**

European ancestry dominated the global evidence base, representing 51.5% of 651,532 participants, while African ancestry accounted for only 0.46% (n = 3,031). Strong correlations were observed among European-leaning Antioquians (*r* ^2^ ≥ 0.90), whereas PLQ exhibited inverse or negligible correlations with those groups (*r* ^2^ = −0.20 to −0.02) and minimal similarity with CHG (*r* ^2^ = 0.12). Twenty-eight SNPs were frequent in PLQ (>75%) but rare in Europeans (<50%), and 44 showed the opposite pattern. Notable examples include CYP3A4 rs3735451-C (rivaroxaban; 87.1% vs. 23.2%), CYP3A5 rs776746-T (tacrolimus; 85% vs. 23.5%), and rs55881666-C (duloxetine; 15% vs. 84%). Globally, 71.5% of PGx studies originated in high-income countries.

**Conclusion:**

Large, clinically actionable allele-frequency contrasts and pronounced discovery biases confirm the need for ancestry-aware PGx testing and locally calibrated dosing algorithms in Colombia. The analytic framework and variant catalogue generated knowledge to operationalize precision pharmacotherapy across admixed Latin-American populations.

## 1 Introduction

Precision pharmacotherapy (the alignment of drug choice and dose with a patient’s genetic makeup), has moved from conceptual promise to clinical necessity as the global burden of non-communicable diseases escalates (Hicks et al., 2019). Pharmacogenomics (PGx) underpins this transition by revealing how germ-line variation modulates pharmacokinetics, pharmacodynamics, efficacy, and toxicity (Cecchin and Stocco, 2020). Yet the clinical translation of PGx discoveries remains uneven because most variant–drug associations were discovered in populations of largely European descent, despite clear evidence that allele frequencies and linkage patterns differ markedly across ancestral groups (Corpas et al., 2024). These differences are not trivial: variant frequency shifts of only a few percentage points can alter the cost-effectiveness of pharmacogenomic testing and, ultimately, population-level outcomes (Karamperis et al., 2024). For regions carrying disproportionate morbidity and mortality, Latin America, sub-Saharan Africa, and parts of South-East Asia in particular, under-representation in PGx research perpetuates therapeutic inequities and limits the external validity of dosing algorithms, clinical guidelines, and decision-support tools (Zhou et al., 2025).

Recognizing this gap, several international roadmaps have made population diversity a core pillar of personalized medicine (The Global Alliance for Genomics and Health, 2025; Ambrosino et al., 2024; National Health Institutes, 2025). The Global Alliance for Genomics and Health. (2025) the World Health Organization’s Genomic Medicine Implementation framework (Ambrosino et al., 2024), and the Precision Medicine Initiative (National Health Institutes, 2025) have all highlighted the need to expand PGx discovery and implementation to low- and middle-income countries (LMICs) (The Global Alliance for Genomics and Health, 2025; Ambrosino et al., 2024; National Health Institutes, 2025). Concurrently, expert working groups such as Clinical Pharmacogenetics Implementation Consortium (CPIC) (Relling and Klein, 2011), Pharmacogene Variation Consortium (PharmVar) (Gaedigk et al., 2021), and the PharmGKB consortium (Thorn et al., 2013) have issued calls for ancestry-aware dosing recommendations, arguing that the absence of local frequency data erodes both the safety and the utility of current guidelines (Relling and Klein, 2011; Gaedigk et al., 2021; Thorn et al., 2013). Evidence syntheses further show that ancestry bias hampers polypharmacy optimization, complicates comparative-effectiveness studies, and obscures variant penetrance estimates in admixed populations (Relling and Klein, 2011; Gaedigk et al., 2021; Thorn et al., 2013). Thus, mapping PGx variation in under-studied settings is now viewed not only as a scientific imperative but as an ethical requirement for equitable healthcare delivery (Relling and Klein, 2011; Gaedigk et al., 2021; Thorn et al., 2013).

Colombia represents a paradigmatic case. Centuries of admixture among Native American, European, and African founders have produced highly stratified genomic ancestries that vary over fine geographic scales, from the predominantly African heritage of San Basilio de Palenque to the predominantly European heritage of Antioquia (Nagar et al., 2019). Allele frequencies extracted from emerging national resources, such as the Consortium for Genomic Diversity, Ancestry, and Health in Colombia (CÓDIGO) (Mariño-Ramírez et al., 2025), illustrate this heterogeneity, yet no systematic effort has linked these data to curated variant–drug annotations. Consequently, Colombian clinicians must extrapolate from frequency tables generated in distant populations, risking misclassification of metabolizer status and inappropriate dosing. For precision-medicine programs that rely increasingly on electronic health-record integrations and pre-emptive genotyping panels, such blind spots undermine both patient safety and health-system efficiency (Chande et al., 2020).

A comprehensive ancestry-stratified PGx landscape would generate immediate translational dividends (Riess et al., 2024). Clinically, it would enable locally calibrated screening panels, and anticipate adverse drug reactions in psychiatry and oncology, therapeutic areas already burdening the national health budget. At the diagnostic level, it would facilitate genetic counselling for monogenic pharmacogenetic disorders (e.g., dihydropyrimidine dehydrogenase deficiency) and guide cascade testing in families (Riess et al., 2024). From a public-health perspective, frequency data stratified by ancestry could inform essential-medicine formularies, ensuring that cost-intensive PGx testing is targeted to medications and populations where the number-needed-to-genotype is lowest (Gemmati et al., 2019). Moreover, an ancestry-aware pharmaco-epidemiologic profile provides a foundation for equitable clinical-trial design, supports pharmacovigilance, and aligns national policy with global precision-medicine benchmarks (Gemmati et al., 2019).

This study aims to integrate all PharmGKB variant–drug annotations with allele-frequency data from five well-characterized Colombian sub-populations to (i) delineate the national pharmacogenomic landscape across ancestries, (ii) identify variants showing the greatest inter-ancestry frequency differentials with direct therapeutic relevance, and (iii) contextualize global research activity through a bibliometric analysis of the studies underlying each annotation. By addressing a critical knowledge gap in an admixed LMIC setting, this work seeks to advance the equitable implementation of clinical pharmacogenomics and to provide a scalable template for other under-represented regions.

## 2 Methods

### 2.1 Study design

Cross-sectional study.

### 2.2 Data collection

We retrieved all available variant drug annotations from the PharmGKB database (University, 2025) on 05 March 2025, by downloading two datasets: one containing the full set of variants–drug annotations, which includes associations related to drug dosage, response, metabolism, among others; and another containing study-level metadata, including population sizes and the biogeographical classification of the study cohorts. These datasets were subsequently merged to create a unified dataset. In total, we identified 4,462 variant annotations reported across 1,225 studies (Supplementary Material 1).

From this initial dataset, we selected annotations that met the following three criteria: a) the variant was a single-nucleotide polymorphism (SNP) reported with a single allele (excluding genotypes and gene variants); b) the association was statistically significant (p < 0.05); and c) an allele frequency was reported. To obtain allele frequencies for these SNPs in Colombian populations, we implemented an automated web query using Python to access the CÓDIGO database (Mariño-Ramírez et al., 2025). Specifically, we extracted allele frequencies for two populations with predominantly African ancestry: Palenque (PLQ, n = 34, 84% African ancestry) and Chocó (CHG, n = 96, 76% African ancestry), and three with predominantly European ancestry from Antioquia: ATQCES (n = 404, 50.5% European Ancestry), ATQPGC (n = 624, 55% European ancestry), and CLM (n = 96, 62.9% European ancestry). These percentages correspond to the average proportion of genetic ancestry fractions inferred with ADMIXTURE in the CÓDIGO database, which estimates contributions from African, Indigenous American, and European sources in Colombian populations. For clarity, we report here only the predominant ancestry fraction for each population, while the complete admixture profiles can be consulted in the original CÓDIGO publication.

Allele frequencies for the PLQ and ATQPGC populations were derived from whole-genome sequencing, whereas those for the remaining populations were obtained via whole-exome sequencing or whole-genome genotyping. Detailed information regarding the number of genomes, exomes, or genotypes analyzed is available in the CÓDIGO article (Mariño-Ramírez et al., 2025), which at the time of data extraction (release 1.0) comprised 1,441 Colombian genomic variant samples across fourteen distinct populations, contributed by investigators from various participating institutions across Colombia. The CÓDIGO database contained 95,254,482 non-redundant variants derived from the merging and harmonization of 123,187,329 high quality genomic variants from eight independent datasets. This filtering process resulted in a refined dataset comprising 1,216 variant annotations reported across 552 studies (Supplementary Material 2), which served as the basis for all subsequent analyses.

### 2.3 Statistical analysis

Using the refined dataset of 1,216 variant annotations, we conducted Spearman’s correlation analyses based on the reported allele frequencies across populations. Variants with missing values were excluded from each pairwise comparison. For ancestry-based comparisons, we calculated the mean allele frequency within each ancestry group: African ancestry was represented by the PLQ population, and European ancestry was defined as the average across the ATQCES, ATQPGC, and CLM populations. Additionally, we classified all individuals included in the original 1,225 studies as either cases or controls, according to the biogeographical ancestry information reported in each study. Participants were categorized as European, Asian, American, African, Other, or Unknown.

### 2.4 Bibliometric analysis

Metadata for each of the 1,225 studies linked to the identified variant annotations was retrieved using the PubMed ID (PMID) through the NCBI Entrez API, Unpaywall API, and Crossref API. Journal metrics (H-index and quartile) were obtained from the SCImago Journal Rank (SJR) databases corresponding to the article’s year of publication (1999–2024). The final dataset included the article title, publication date, number of authors, first author’s country of affiliation, access status (open or closed), citation count, and the year-specific H-index and quartile. The first author’s country of affiliation was used to assign each study to a World Health Organization, 2025 (WHO) region (World Health Organization) and a World Bank income classification (World Bank, 2025), which served as key variables in the descriptive bibliometric analysis.

This bibliometric mapping was crucial to contextualize population-level allele frequency disparities within the global landscape of PGx research. By cross-referencing allele frequencies with study-level metadata, we ensured that the frequency contrasts observed in Colombian populations aligned with the clinical significance and evidentiary strength of the associated variant–drug annotations.

All the analyses were conducted in R (v4.4.0)⁠. The script and datasets, along with detailed annotations, are available at https://doi.org/10.5281/zenodo.15361131.

## 3 Results

### 3.1 Baseline characteristics of the studies

**FIGURE 1:**
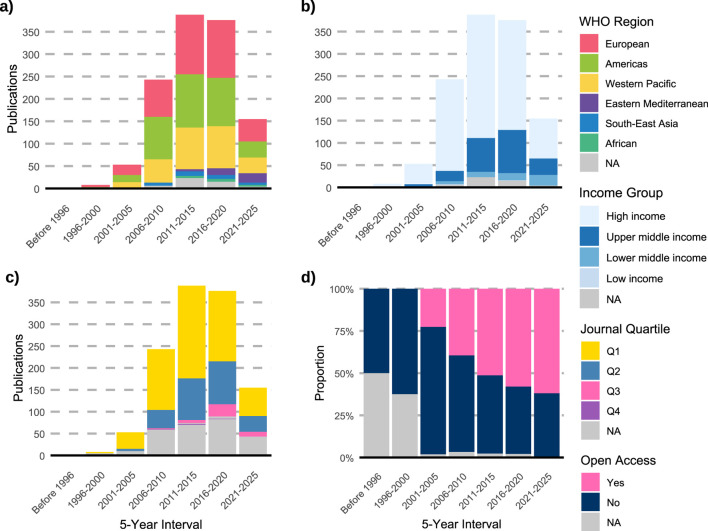
Temporal trends and characteristics of pharmacogenomics studies indexed in PharmGKB (n = 1,225). **(a)** Distribution of publications by WHO region across 5-year intervals. **(b)** Distribution of studies by World Bank income group. **(c)** Distribution of publications by journal quartile (Q1–Q4). **(d)** Proportion of studies published as open access over time. NA: not available.

All 4,462 variants identified in the first step of the research methodology were reported across 1,225 studies, representing all studies of variant drug annotations registered in PharmGKB. The earliest of these publications’ dates to 1994, appearing in Pharmacogenetics and Genomics journal, and described an association between the T allele and reduced warfarin metabolism compared to the C allele (Rettie et al., 1994). Of the total studies, 34.5% originated from the European region and 30.7% from the Americas (Figure 1a; Table 1) while 71.5% were conducted in high-income countries (Figure 1b; Table 2). These same regions contributed the majority of citations: 27,351 (38.8%) from the Americas, 25,501 (36.2%) from Europe, and 61,432 (87.2%) from high-income countries. The highest open access ratios were observed in the Americas (2.34) and in high-income countries (1.19) (Tables 1, 2). In terms of journal ranking, 65.3% of articles were published in Q1 journals (Figure 1c), although 50.7% of all articles were not open access (Figure 1d).

**TABLE 1:** Baseline characteristics of publications on pharmacogenomics by region (N = 1,225).

Variable	Americas	Europe	Western Pacific	South-East Asia	Eastern Mediterranean	Africa	Not retrieved
Publications (%)	376 (30.7)	424 (34.6)	289 (23.9)	31 (2.53)	41 (3.35)	16 (1.31)	48 (3.92)
Total citations (mean per paper)	27,351 (72.7)	25,501 (60.1)	13,334 (46.1)	669 (21.6)	337 (8.22)	394 (24.6)	2,855 (59.5)
Median H-index (IQR)	154 (145)	154 (114)	118 (80)	126 (78.5)	103 (81.2)	154 (61.8)	152 (70.5)
Open access/No open access (ratio)	262/112 (2.34)	190/223 (0.85)	105/173 (0.61)	10/20 (0.5)	20/17 (1.18)	7/9 (0.78)	27/19 (1.42)
Journal quartile (%) (n = 946)							
Q1	205 (71.18)	209 (67.42)	139 (57.92)	12 (52.17)	10 (34.48)	10 (71.43)	33 (78.57)
Q2	75 (26.04)	86 (27.74)	86 (35.83)	10 (43.48)	13 (44.83)	4 (28.57)	2 (4.76)
Q3	8 (2.78)	14 (4.52)	14 (5.83)	0	6 (20.69)	0	6 (14.29)
Q4	0	1 (0.32)	1 (0.42)	1 (4.35)	0	0	1 (2.38)

**TABLE 2:** Baseline characteristics of publications on pharmacogenomics by income group (N = 1,225).

Variable	High income	Upper-middle income	Lower-middle income	Low-income	Not retrieved
Publications (%)	876 (71.5)	239 (19.5)	59 (4.82)	3 (0.25)	48 (3.9)
Total citations (mean per paper)	61,432 (87.5)	5,067 (7.19)	938 (1.33)	149 (0.21)	2,855 (4.05)
Median H-index (IQR)	154 (114)	106 (69.8)	126 (80.5)	140 (14)	125 (70.5)
Open access/No open access (ratio)	468/394 (1.19)	100/126 (0.79)	24/33 (0.73)	2/ 1 (2)	27/19 (1.42)
Journal quartile (%) (n = 946)					
Q1	460 (70.4)	104 (50.2)	19 (45.2)	2 (100)	33 (78.6)
Q2	175 (26.8)	83 (40.1)	16 (38.1)	0	2 (4.8)
Q3	18 (2.8)	18 (8.7)	6 (14.3)	0	6 (14.3)
Q4	0	2 (1)	1 (2.4)	0	1 (2.4)

### 3.2 Genetic ancestry plays a key role in shaping pharmacogenomic profiles in Colombia

**FIGURE 2:**
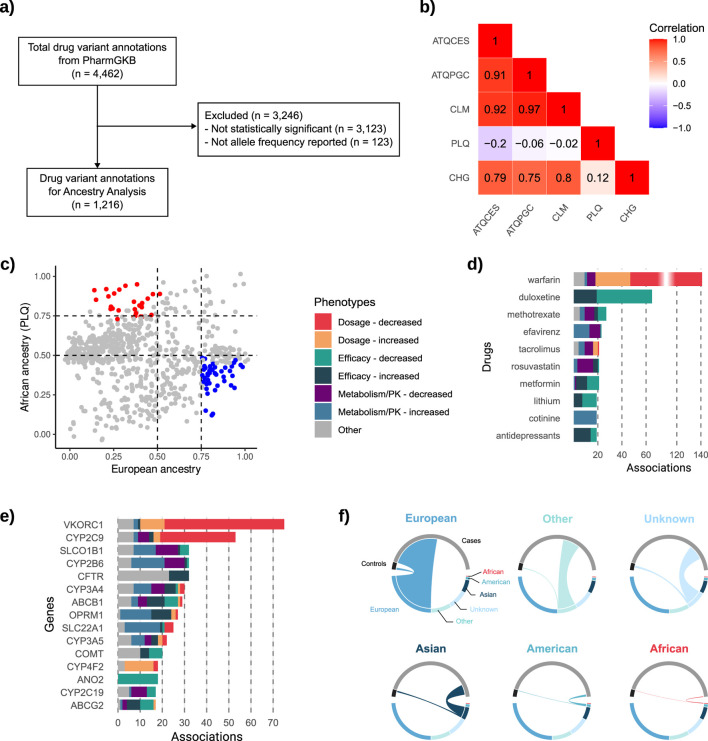
Ancestry representation, pharmacogenomic variant selection, and distribution of drug- and gene-level associations. **(a)** Flowchart of the inclusion criteria applied to drug variant annotations retrieved from PharmGKB (n = 4,462), resulting in 1,216 annotations. **(b)** Heatmap showing Spearman’s correlation coefficients for allele frequencies across five Colombian populations: ATQCES, ATQPGC, CLM, PLQ (San Basilio de Palenque), and CHG (Chocó). **(c)** Scatterplot of allele frequencies in PLQ (y-axis, African ancestry) versus the mean allele frequency of ATQCES, ATQPGC, and CLM (x-axis, European ancestry) for all significant variants. **(d)** Top 10 drugs with the highest number of significant associations, categorized by phenotype. **(e)** Top 15 genes with the most reported associations, color-coded by phenotype. **(f)** Chord plots showing the ancestry composition of individuals included in the 1,225 pharmacogenomic studies, grouped by ancestry category and study role (cases vs. controls).

Using the 1,216 variant annotations that met the inclusion criteria (Figure 2a), we first performed Spearman’s correlation analysis. Correlation coefficients were notably strong among Colombian populations with predominantly European ancestry (ATQCES, ATQPGC, and CLM), each exhibiting value ≥ 0.9, indicating highly similar PGx profiles in terms of allele frequency (Figure 2b). In contrast, the Colombian population with the highest proportion of African ancestry, San Basilio de Palenque, displayed markedly different PGx profiles compared to the predominantly European populations, which each have less than 13% African ancestry (ATQCES: *r* ^2^ = −0.20; ATQPGC: *r* ^2^ = −0.06; CLM: *r* ^2^ = −0.02). Notably, San Basilio de Palenque also showed low similarity with Chocó (*r* ^2^ = 0.12), a population with predominantly African ancestry but higher European admixture (11.5%). Chocó, in turn, exhibited moderate positive correlations with the European ancestry populations (ATQCES: *r* ^2^ = 0.79; ATQPGC: *r* ^2^ = 0.75; CLM: *r* ^2^ = 0.80), although to a lesser extent.

Given the unique PGx profile of San Basilio de Palenque compared to other populations, we identified the predominant alleles in this population relative to European populations. To facilitate comparison, we calculated the mean allele frequency across the ATQCES, ATQPGC, and CLM populations (chosen due to their high pairwise correlation), and plotted these values against the allele frequencies observed in PLQ for all significant SNPs (Figure 2c). This analysis revealed 28 SNPs with high frequency in PLQ (allele frequency >75%) and low frequency in European populations (mean allele frequency <50%). Conversely, 44 SNPs were more frequent in European populations (mean allele frequency >75%) and less common in PLQ (allele frequency <50%). Most of these SNPs were associated with either efficacy phenotypes or metabolism/pharmacokinetics traits. Here, we report a subset of these variants with the largest differences in allele frequency between groups (Table 3); the full list is available in Supplementary Material 3.

**TABLE 3:** Pharmacogenomic variants with differential allele frequencies by ancestry and their associated phenotype and drug effects.

Gene	SNP	Drug	Phenotype and direction of effect	PLQ (AF%)	European (AF%)	Reference
SNPs most predominant in San Basilio de Palenque						
*CYP3A4*	rs3735451-C	Rivaroxaban	Metabolism/PK increased	87.1	23.23	Li et al. (2024)
*CYP3A5*	rs776746-T	Tacrolimus	Dosage, Metabolism/PK increased	85	23.5	Kim et al. (2012)
*CDKAL1*	rs7754840-C	DPP-4 inhibitors	Efficacy increased	76.1	28.4	Osada et al. (2016)
SNPs most predominant in European populations						
Not retrieved	rs55881666-C	Duloxetine	Efficacy decreased	15	84	Maciukiewicz et al. (2018)
*PTPRC*	rs10919563-G	Adalimumab, etanercept, infliximab	Efficacy increased	28	75.6	Cui et al. (2010)
*ABCB1*	rs7787082-G	Clozapine	Efficacy decreased	29	79.3	Lee et al. (2012)

AF, allele frequency; PLQ, san basilio de palenque; SNPs, Single Nucleotide Polymorphisms.

Among the predominant SNPs in the PLQ population, rs3735451-C (associated with increased rivaroxaban concentrations in individuals with atrial fibrillation (Li et al., 2024)), was present in 87.1% of PLQ individuals, compared to only 23.2% in European populations. Similarly, rs776746-T, linked to enhanced metabolism of tacrolimus in kidney transplant recipients (Kim et al., 2012), was found in 85% of PLQ individuals and 23.5% of Europeans. Additionally, rs7754840-C, associated with improved response to dipeptidyl peptidase-4 (DPP-4) inhibitors in people with diabetes mellitus (Osada et al., 2016), had a frequency of 76.1% in the PLQ population and 28.4% in European populations (Table 3).

Conversely, in European populations, rs55881666-C (associated with reduced response to duloxetine in major depressive disorder (Maciukie et al., 2018)), was present in 84%, but only 15% of PLQ individuals. rs10919563-G, linked to increased response to adalimumab, etanercept, or infliximab in rheumatoid arthritis (Cui et al., 2010), showed a frequency of 75.6% in Europeans and 28% in PLQ. Finally, rs7787082-G, associated with decreased response to clozapine in schizophrenia (Lee et al., 2012), was present in 79.3% of Europeans and 29% of PLQ individuals (Table 3).

What is being studied in pharmacogenomics worldwide?

From the total number of associations retrieved (Figure 2a), we also examined: a) the drugs most frequently studied over time, and b) the genes with the greatest number of reported associations. We found that the top 10 drugs with the most significant associations (Figure 2d), according to our selection criteria (Figure 2a), accounted for 30.9% of all identified associations. Warfarin emerged as the most studied drug, representing 11.5% of all associations, followed by duloxetine (5.3%) and methotrexate (2.2%). Among these drugs, the most commonly associated phenotypes were related to decreased dosage (25.2%) and decreased efficacy (21.8%). Regarding genes, 36.7% of all associations involved the top 15 genes shown in Figure 2e. *VKORC1* had the highest number of associations (6.1%), followed by *CYP2C9* (4.3%), and *SLCO1B1*, *CYP2B6*, and *CFTR* (each representing 2.6%). Among these top genes, the most frequently reported phenotype was decreased dosage.

Importantly, several of these top-ranked genes such as *CYP3A4*, *CYP3A5*, *VKORC1*, and *SLCO1B1*, were also among those showing significant allele frequency differentials across Colombian populations, underscoring their clinical relevance for pharmacogenomic implementation in the country.

### 3.3 The European bias

A total of 651,532 individuals were included as either cases (n = 609,280; 93.6%) or controls (n = 42,252; 6.4%) across the 1,225 studies retrieved through our PharmGKB-based search strategy. Of these, 336,073 individuals (51.5%) were of European ancestry, comprising the largest proportion (Figure 2f). Participants of Asian and American ancestry accounted for 72,359 (11.1%) and 14,470 (2.22%), respectively. And only total of 3,031 individuals (0.46%) were identified as being of African ancestry. The remaining participants either had unreported ancestry (n = 110,073; 16.8%) or were classified as having other ancestry (n = 115,526; 17.7%) (Supplementary Material 1).

## 4 Discussion

The present study offers the first ancestry-stratified, variant-level PGx landscape for Colombia and, by extension, one of the most comprehensive illustrations of how global discovery pipelines still falter at the point of regional implementation. By integrating 1,216 statistically significant PharmGKB annotations with allele-frequency data from five Colombian sub-populations, we were able to interrogate three complementary layers of evidence (bibliometrics, ancestry representation, and population genetics), and evaluate whether current knowledge is fit for purpose in a highly admixed Latin-American context (Nagar et al., 2019).

Global discovery still follows the money and the map. Of the 1,225 publications that define every PharmGKB variant–drug annotation, 71.5% were generated in high-income countries, and a combined 65.3% appeared in Q1 journals; yet only half of all articles were open access, with the lowest openness ratios in lower-middle-income settings. Although the Americas contributed almost one-third of the evidence base, the distribution of first authors and citation density still mirrors the traditional North–South gradient (Lozada-Martinez et al., 2024a). These findings were expected, bibliometric audits have repeatedly shown that resource-rich settings dominate PGx output, but the magnitude of the skew highlights a persistent implementation gap: the loci most likely to inform therapeutic decisions in LMICs are the least likely to have been discovered, replicated, or translated locally (Karamperis et al., 2024; Zhou et al., 2025). The 2.34 open-access ratio for the Americas is a relative bright spot, suggesting regional investigators may be more proactive in disseminating data without paywalls; nevertheless, gated evidence remains a barrier for national pharmacovigilance agencies and formularies (E and lse, 2024).

Ancestry bias remains the Achilles’ heel of clinical PGx (Corpas et al., 2024). Across 651,532 phenotype-genotype records, 51.5% of participants were of European ancestry, whereas only 0.46% were classified as African and 2.22% as American (Native/admixed). In a country such as Colombia, whose demographic history is defined by tri-hybrid admixture (Nagar et al., 2019), this imbalance is more than academic: every dosing recommendation imported from European-centric studies carries unquantified risk. The fact that nearly 17% of individuals in the global dataset had unreported ancestry underscores an avoidable source of misclassification that can propagate through meta-analyses and guideline panels. While the direction of the bias is unsurprising, its persistence in 2025, despite repeated calls for equity in discovery science (The Global Alliance for Genomics and Health, 2025; Ambrosino et al., 2024; National Health Institutes, 2025), signals that conventional funding and peer-review incentives are insufficient to correct course.

Fine-scale correlations reveal a bifurcated Colombian PGx landscape. Correlation analyses showed near-identity among the three Antioquian populations (ATQCES, ATQPGC, CLM; *r* ^2^ ≥ 0.90), confirming the intuitive expectation that closely related founder histories yield highly similar PGx profiles. Conversely, PLQ (the settlement with the highest African ancestry) displayed either weakly negative or negligible correlations with every European-leaning group (*r* ^2^ = −0.20 to −0.02) and only minimal concordance with Chocó, another Afro-descendant population (*r* ^2^ = 0.12).

That lack of correlation between PLQ and Chocó was not fully expected: both communities share African roots, yet founder effects, differential gene flow, and local selection pressures appear to have sculpted distinct allele spectra. For clinicians, these results caution against blanket use of “African-ancestry” modifiers in dosing algorithms; intra-continental diversity can be as large as inter-continental diversity at clinically actionable loci (Munung, 2025).

Variant-level differentials translate into tangible therapeutic risk or benefit. We identified 28 SNPs common in PLQ but rare in European Colombians, and vice versa for 44 SNPs. Three illustrate the point. The rivaroxaban-associated *CYP3A4* variant rs3735451-C occurs in 87.1% of PLQ residents versus 23.2% of Europeans, predicting higher drug exposure and a potential bleeding-risk differential (Baturina et al., 2023). These gene–drug associations are especially relevant in Colombia, where cardiovascular disease and organ transplantation are growing public health priorities. For example, tacrolimus is a cornerstone immunosuppressant in Colombian transplant programs, and rivaroxaban is increasingly prescribed for atrial fibrillation (Chande et al., 2020). Thus, population-specific pharmacogenomic profiling of these variants has immediate clinical and policy implications (Chande et al., 2020).

The tacrolimus metabolism enhancer rs776746-T in *CYP3A5* shows a similar 85% vs. 23.5% split, affirming prior data that Afro-descendant transplant recipients may require higher doses to reach target trough levels (Genvigir et al., 2020). Conversely, the duloxetine efficacy-reducing rs55881666-C is fourfold more prevalent in Europeans (84% vs. 15%), implying that dose-escalation strategies validated in European cohorts might be ineffective or even harmful in PLQ patients (Maciukiewicz et al., 2015).

Such magnitudes of difference were unlikely to have been predicted without empirical frequency mapping and justify pre-emptive genotyping in high-contrast settings.

What is studied is not what Colombia needs most. Warfarin, duloxetine, and methotrexate dominate the global annotation landscape, collectively representing nearly one-fifth of all significant associations; *VKORC1* and *CYP2C9* lead the gene rankings.

While warfarin remains clinically relevant, rivaroxaban and other direct oral anticoagulants have gained market share in Colombia; duloxetine, though important, does not account for the majority of antidepressant prescribing (Machado-Duque et al., 2021). The mismatch suggests that local disease burden and drug-utilization patterns are poorly aligned with global PGx discovery priorities, echoing critiques that pharmacogenomics has yet to pivot from “variant hunting” to “clinical-need hunting” (El-Gowilly et al., 2024).

Non-significant results are equally informative. Moderate correlations between Chocó and European populations (*r* ^2^ = 0.75–0.80) did not reach the stringent thresholds applied to designate high similarity, yet they indicate partial allele sharing likely driven by historic admixture.

These borderline results matter because they caution against dichotomizing ancestry as African versus European; instead, decision-support tools should incorporate continuous local ancestry or probabilistic genotype imputation. Likewise, two-thirds of PharmGKB variants retrieved for Colombia failed to meet the p < 0.05 significance filter, underscoring how many published associations remain under-powered or population-specific. This is a reminder that the absence of evidence is not evidence of absence (Feres and Feres, 2023) but a prompt for replication consortia.

Taken together, these findings strengthens the premise that ancestry-aware PGx panels are essential for equitable precision medicine programs (Nagar et al., 2020). For health-technology-assessment bodies, the allele-frequency contrasts we document may feed directly into cost-effectiveness models by refining the number-needed-to-genotype and number-needed-to-treat (Picón-Jaimes, 2024m). For drug-regulatory agencies, the European participant over-representation highlights the need for post-marketing surveillance in admixed populations, particularly when authorizing drugs with narrow therapeutic indices. National research councils can leverage the bibliometric data to negotiate open-access mandates with journals or funder-publisher agreements, accelerating the flow of locally relevant evidence.

### 4.1 Knowledge gaps as research opportunities

The pronounced scarcity of African-ancestry data, even within Latin America, presents an immediate agenda for collaboration with Afro-Colombian communities, prioritizing co-created protocols that address historical mistrust. Comparative PGx trials that stratify by fine-scale local ancestry could serve as a template for other admixed nations. Integrating electronic health records with prospective biobanks in Colombia would enable rapid-learning healthcare systems where allele-frequency updates continuously refine clinical-decision-support algorithms (Picón-Jaimes, 2024m).

To accelerate the clinical translation of these findings, Colombia’s Ministry of Health and scientific institutions could prioritize the development of ancestry-calibrated dosing algorithms, integrate PGx alerts into electronic health records, and fund replication studies in high-risk populations such as Afro-Colombians. Building trust and co-designing protocols with underrepresented communities will be essential for ethical and sustainable implementation.

### 4.2 Contribution to precision medicine and genetic medicine

By juxtaposing global evidence with Colombian genetic diversity, this study highlights a critical data gap and provides a scalable analytic framework for other LMICs. It furnishes actionable variant lists for immediate incorporation into laboratory-developed tests, informs guideline writers about population-specific effect sizes, and equips payers with the epidemiologic inputs needed to justify reimbursement for PGx screening (Tamraz et al., 2025; Lozada-Martinez et al., 2024b; Lozada-Martinez et al., 2025a; Lozada-Martinez et al., 2025b; Lozada-Martinez et al., 2025c). In doing so, it advances the regional transition from one-size-fits-all therapeutics toward ancestry-calibrated care pathways that can improve efficacy, safety, and cost-effectiveness across Latin America (Lozada-Martinez et al., 2024a; Pérez-Fontalvo et al., 2021).

### 4.3 Limitations and future directions

The objective of this work was not to re-estimate pharmacogenomic effects, but rather to conduct a meta-research exercise that translates literature-validated pharmacogenomic associations into an ancestry-resolved, population-specific context for Colombia. The associations analyzed were curated from peer-reviewed primary studies that reported clearly defined clinical endpoints. By integrating these associations with harmonized allele frequency data from the CÓDIGO database, we aimed to identify ancestry-specific variants of potential clinical relevance. Therefore, our findings should be considered hypothesis-generating rather than causal, and we explicitly call for prospective validation in Colombian cohorts using individual-level genomic data linked to longitudinal clinical outcomes. Such validation studies would bridge the current gap between population-level allele frequency differentials and individual patient care outcomes.

## 5 Conclusion

This study delivers, to our knowledge, the first ancestry-resolved pharmacogenomic map of Colombia, integrating all PharmGKB variant–drug annotations with allele-frequency data from five genetically distinct sub-populations. First, it was demonstrated that European-derived evidence dominates the global PGx knowledge base, leaving critical dose-modifying loci under-studied in admixed and African-ancestry Colombians. Second, it was quantified clinically meaningful inter-ancestry frequency differentials (for example, the CYP3A5*3 enhancer rs776746-T is four times more common in San Basilio de Palenque than in European-leaning Antioquia), signaling a tangible need for ancestry-informed dosing of tacrolimus, rivaroxaban, and duloxetine. Third, the bibliometric audit exposes persistent geographic and access barriers that limit LMIC investigators’ ability to validate, replicate, and implement PGx discoveries.

These insights equip policymakers and payers with the empirical inputs (allele frequencies, effect sizes, and evidence gaps) needed to model the cost-effectiveness of pre-emptive genotyping and to prioritize reimbursement for high-impact tests. Regulators can use our variant catalogue to refine pharmacovigilance triggers and to mandate post-marketing surveillance in high-risk sub-groups. For researchers, the dataset highlights under-represented alleles and populations ripe for replication studies, multi-center trials, and guideline development. More broadly, this analytic framework is immediately transferable to other admixed LMICs, advancing the global agenda for equitable precision medicine.

## Data Availability

The original contributions presented in the study are included in the article/Supplementary Material, further inquiries can be directed to the corresponding authors.
